# Channel Characteristics and Link Adaption for Visible Light Communication in an Industrial Scenario

**DOI:** 10.3390/s23073442

**Published:** 2023-03-24

**Authors:** Yu Tong, Pan Tang, Jianhua Zhang, Shuo Liu, Yue Yin, Baoling Liu, Liang Xia

**Affiliations:** 1 State Key Laboratory of Networking and Switching Technology, Beijing University of Posts and Telecommunications, Beijing 100876, China; 2 China Mobile Research Institute, Beijing 100053, China

**Keywords:** the sixth generation (6G), visible light communication (VLC), industrial internet of things (IIoT), channel characteristics, large-scale fading, multipath-related characteristics, link adaption method

## Abstract

Visible light communication (VLC) is one of the key technologies for the sixth generation (6G) to support the connection and throughput of the Industrial Internet of Things (IIoT). Furthermore, VLC channel modeling is the foundation for designing efficient and robust VLC systems. In this paper, the ray-tracing simulation method is adopted to investigate the VLC channel in IIoT scenarios. The main contributions of this paper are divided into three aspects. Firstly, based on the simulated data, large-scale fading and multipath-related characteristics, including the channel impulse response (CIR), optical path loss (OPL), delay spread (DS), and angular spread (AS), are analyzed and modeled through the distance-dependent and statistical distribution models. The modeling results indicate that the channel characteristics under the single transmitter (TX) are proportional to the propagation distance. It is also found that the degree of time domain and spatial domain dispersion is higher than that in the typical rooms (conference room and corridor). Secondly, the density of surrounding objects and the effects of user heights on these channel characteristics are also investigated. Through the analysis, it can be observed that the denser objects can contribute to the smaller OPL and the larger RMS DS under the single TX case. Furthermore, due to the blocking effect of surrounding objects, the larger OPL and the smaller RMS DS can be observed at the receiver with a low height. Thirdly, due to the distance dependence of the channel characteristics and large time-domain dispersion, the link adaption method is further proposed to optimize the multipath interference problem. This method combines a luminary adaptive selection and delay adaption technique. Then, the performance of the link adaption method is verified from four aspects through simulation, including the signal-to-noise (SNR), the RMS DS, the CIRs, and the bit-error rate (BER) of a direct-current-biased optical orthogonal frequency division multiplexing (DCO-OFDM) system. The verification results indicate that our proposed method has a significant optimization for multipath interference.

## 1. Introduction

With the increasing demands for extremely high capacity and emerging applications for the sixth generation (6G) networks, the over-crowded radio frequency (RF) may not meet the considerable increasing needs of 6G [1]. To address these issues, visible light communication (VLC) has emerged as a potential technology that integrates communication and illumination [2]. The health-friendly, low-cost, and energy-conserved VLC can provide a high transmission rate and strong resistance to electromagnetic interferences in its unlicensed broad available spectrum (400–800 THz) [3]. Based on the simplicity and ubiquity of such advantages, VLC, also referred to as LiFi, has been utilized for many communication tasks in the Industrial Internet of Things (IIoT) [4], which suffers from interference and electromagnetic noise produced by metal machinery.

The ultimate performance limits of a communication system are determined by the channel in which it operates [5]. VLC channel modeling is the foundation of designing and optimizing VLC communication systems, which can evaluate the performance limit of the VLC system [6]. Therefore, the realistic channel models for IIoT scenarios are of critical importance to accurately predict the propagation characteristics. There are already indoor VLC channel models in existing standards. However, the IIoT scenario has a larger physical size, with many more metal machine tools and production lines in it [7] than in an indoor office. Thus, the IIoT scenario exhibits many new channel characteristics, e.g., a dense multipath reflected by the abundance of metallic scatterers. Moreover, the light rays cannot penetrate the walls and will experience greater blocking caused by the equipment during propagation. Hence, modeling the VLC channel in the IIoT scenario is necessary for industrial applications, and the existing channel models for typical indoor scenarios are not applicable in factories [7]. In addition, the channel characteristics (e.g., path loss and delay spread) might vary significantly [5] due to the larger physical size in the IIoT scenario. Furthermore, the severe multipath fading caused by the scatterers will also contribute to inter-code interference (ICI), leading to the difficulty of the VLC communication system. Therefore, a link adaptation method should be proposed to optimize the multipath interference.

This manuscript mainly focuses on the VLC channel characterization in the IIoT scenario and the designing of the link adaption method. In the state of the art, several works have been conducted on these two aspects. In terms of VLC channel modeling in indoor scenarios, the works can be divided into two groups, i.e., simulations and measurements. In the first group, theoretical VLC channel models (e.g., recursive and iterative models) and ray-tracing simulations are usually used to characterize indoor VLC channels. For example, the optical path loss (OPL) of non-Line-of-sight (NLOS) components with different reflection orders was analyzed in [8] by using simulations with the iterative model. Other configurations, e.g., the room size and transceiver deployment, were also considered in [9] through simulations. However, some simplifying assumptions such as empty rooms and fixed reflectance were considered in these works.

Therefore, the most realistic indoor VLC channel modeling is presented in [10,11], where accelerated ray tracing features of Zemax® are used to obtain channel impulse responses (CIRs) for various indoor environments. In [12], the VLC channel characteristics based on the flexible OLED are investigated in shopping malls by using ray-tracing simulations. Numerical models for the delay spread and optical path loss are derived, which followed a 2-term power series model for both empty and furnished rooms. Authors in [13] propose a realistic channel model for VLC assuming a mobile user. The CIRs for each point over the user movement trajectories are obtained and the path loss and delay spread are further proposed as a function of distance through curve fitting. Furthermore, a ray tracing-based channel modeling method is adopted to characterize the effect of user mobility on the VLC channel parameters in [14]. Statistical models for path loss and RMS DS are further proposed in the simulated 3D environments, where the user, upon which three MBSN nodes are placed, walks over extensive realistic random trajectories. Authors in the [15] compared the propagation characteristics between mmWave and VLC bands in a conference room by using ray-tracing simulations. The OPL model was further proposed as a function of the distance and physical size of photodetectors (PDs). In [11], four different real-life-based hospital scenarios were designed to model and characterize the downlink VLC-based MBSN channels. Generally, simulation results should be validated by experimental investigations. In the second group, [16] presented channel measurement campaigns with an empty room, including LOS and NLOS cases. Then, the large-scale OPL and channel impulse responses were investigated. In [17], the wavelength dependence of the optical path loss was experimentally analyzed and modeled in the conference room and corridor.

In terms of the designing of the link adaption method, authors in [13] proposed a simple adaptive VLC transmission scheme that selects both optimal combinations of light-emitting diodes (LEDs) and modulation orders to maximize the signal-to-noise (SNR) and spectral efficiency (SE), while satisfying a targeted value of the bit-error-rate (BER). Furthermore, in [18], the effects of the user-mobility were mitigated by an adaptive VLC transmission technique, which varies the modulation order under a specified BER constraint. An adaptive modulation scheme that aims to maximize the data rate under the constraint of a targeted bit error rate was also considered in [5]. However, these methods cannot solve the problem of the co-channel interference (CCI) under multiple LED cases. Hence, in [19], a novel delay adaptation technique is proposed to mitigate CCI. The authors controlled the time of transmitting the effective signal so that the LOS components from variable transmitters arrive at the receiver side at the same time.

Generally, the existing works regarded to the indoor VLC channel characterization are mainly limited to typical indoor scenarios, such as the conference room, the living room, and the underground mining environment. To the best of our knowledge, almost no existing literature focuses on characterizing and modeling the VLC channel characteristics in the IIoT scenarios. In addition, the selection algorithms of existing link adaption techniques are basically through brute-force search, with high algorithmic complexity. Furthermore, these techniques lack in solving the distance adaption and multipath interference problems simultaneously.

In this paper, the VLC channel characteristics in the IIoT scenarios, including large-scale fading and multipath-related characteristics, are analyzed based on the ray-tracing simulation. Moreover, a two-step approach to optimizing the multipath interference is further proposed. The main contributions can be listed as follows:

- Based on the ray-tracing simulation, the large-scale fading and multipath-related characteristics, including the channel impulse response (CIR), the optical path loss (OPL), and the root mean square (RMS) delay spread (DS), are analyzed and modeled through the distance-dependent and the statistical distribution models. Through analyzing the results, the channel characteristics are dependent on the propagation distance under the single transmitter (TX). Furthermore, the degree of the time domain and spatial domain dispersion in the IIoT scenario is higher than that in the typical indoor environments (e.g., conference room and corridor).
- The effect of the density of surrounding objects on the VLC channel characteristics is analyzed under the single TX. Furthermore, the large-scale fading and multipath-related characteristics at different user heights are compared under the multiple TX cases. We find that the user surrounded by the denser objects can receive much more multipath components, leading to the smaller OPL and larger RMS DS. Furthermore, the larger OPL and the smaller RMS DS can be observed at the receiver (RX) with a low height.
- The link adaption method is further proposed to optimize a multipath interference, which leads to the ICI. The luminary adaptive selection and delay adaption techniques are both combined in the method. Four parameters, including the SNR, the RMS DS, the CIRs, and the BER of the direct-current-biased optical orthogonal frequency division multiplexing (DCO-OFDM) system are simulated to verify the performance of the link adaption method. The verification results indicate that our proposed method has a significant optimization for multipath interference.

The rest of this paper is organized as follows. Section 2 introduces the simulation setup, including the simulation scenario and DCO-OFDM system. The analysis and modeling of the VLC channel characteristics in the IIoT scenario are analyzed and modeled in Section 3. The link adaption method and the verification results are shown in Section 4.

## 2. Simulation Setup

### 2.1. Simulation Scenario Set Up

Since the existing visible channel measurement platform is limited by the LED bandwidth, the complete channel multipath information cannot be accurately obtained. The non-sequential ray tracing features of Zemax have further been used by M. Uysal et al. [10]. VLC channel models developed by this ray tracing method were accepted as reference channel models in IEEE 802.15.13 and IEEE 802.11bb [20]. Furthermore, this channel model approach has been demonstrated by conducting several experimental results [21].

**Figure 1:**
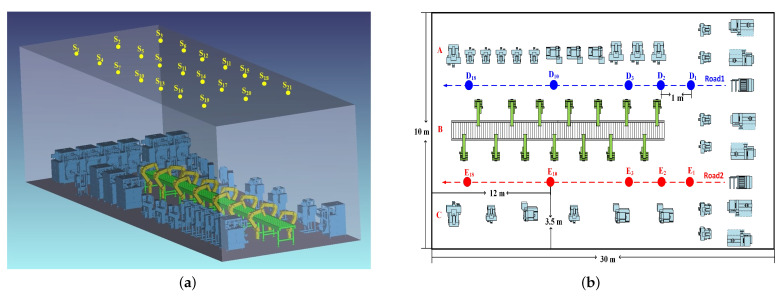
(**a**) The 3D IIoT simulation scenario; (**b**) The layout of the IIoT scenario.

In this work, this well-established ray-tracing channel modeling approach was constructed to investigate the channel characteristics in IIoT scenarios. Firstly, as shown in Figure 1a, the three-dimension (3D) indoor environments with a size of 30×10×8 $m^{3}$ were created as per the requirements in [7]. Then, computer-aided design (CAD) models, such as the floor, wall, manufacturing equipment, assembly lines, forklifts, etc., were imported to model the realistic IIoT scenarios (e.g., object groups A, B, and C shown in the Figure 1b.). The average height of objects set in the scenario is 2 m. Then, the coating materials of walls, manufacturing equipment, assembly lines, and forklifts were, respectively, assumed to be plaster, Copper (Cu), Aluminium (Al), and Ferrum (Fe). The wavelength-dependent spectral reflection characteristics of the surface materials were also integrated into the simulation platform, which can be downloaded from [22]. Moreover, the mixed reflections including diffuse and specular reflections were set by adjusting the scatter fraction (SF) parameter. The specifications of the reference scenario and coating materials of objects under consideration are summarized in Table 1. Twenty-one luminaries ($S_{1}$,..., $S_{21}$), which are commercially available LEDs with a $60^{\circ }$ half viewing angle are placed across the ceiling with equal spacing. We set the optical power at 10 Watts for each luminaire to achieve the average illumination level which satisfies typical illumination levels for an IIoT environment. Moreover, to investigate the omnidirectional channel parameter information of the IIoT scenario, we prefer a spherical photodetector (PD) in this study to collect all the MPCs arriving at the PD as the mmWave antenna does. The field of view (FOV) and the detector size are $360^{\circ }$ and 1 cm${}^{2}$, respectively.

Furthermore, this paper mainly focuses on investigating the distance dependence and the effect of objects on VLC channel characteristics. Therefore, as the layout of the IIoT scenario depicted in Figure 1c, a 3D simulation was constructed in the previous subsection with two specific roads (road1 and road2) that were designed to incorporate different configurations. Eighteen different locations are considered for PDs ($D_{1}$, ..., $D_{18}$) in Road 1. We first modeled the user with a height of 1.5 m (lower than the average height of objects) and 2.5 m (higher than the average height of objects) to study the effect of different user heights on channel characteristics. Furthermore, to investigate the effects of surrounding objects’ densities, we varied the distribution densities of objects surrounding the roads. The same number of points was also considered in Road 2 with a red color ($E_{1}$,..., $E_{18}$). Based on this simulation setting, the influence of various factors on the channel characteristics can be investigated. In our simulation, the Monte Carlo analysis and sobel sampling are adopted as the random ray tracing methods. In addition, the main simulated parameters in the adopted methods are the number of reflections and the number of rays. These parameters can be adjusted through “Relative Minimum Intensity” [23], where the normalized intensity dropped to $10^{-3}$. Then, the non-sequential ray tracing features of Zemax${}^{®}$ are used to calculate the received optical power and path lengths from the TX to the RX for each ray.

### 2.2. DCO-OFDM System Setup

In DCO-OFDM, the binary information is first mapped as the M-order quadrature amplitude modulation (QAM). Then, $N_{p}$ pilots are inserted for channel state information estimation and equalization at the receiver. To ensure that the output of the inverse fast Fourier transforms (IFFT) is real-valued, Hermitian symmetry is added to the signal [24]. After the N-point IFFT operation where the output is denoted by $x_{s}\left[N\right]$, a cyclic prefix with length $N_{CP}$, greater than or equal to the delay spread of the channel (L), is appended to the beginning of each DCO-OFDM frame and parallel streams are converted to serial. Then, the modulated signal is direct-current (DC)-biased for the constraint of IM/DD and transmitted via LED. The time-domain-received signal at photodetector (PD) is [24]:

(1) $$y\left(t\right)=x_{s}\left(t\right)*h_{VLC}\left(t\right)+v_{n}\left(t\right),$$

where $v_{n}\left(t\right)$ is the additive White Gaussian noise (AWGN) with zero means and $\sigma_{N}^{2}$ = $N_{0}W$ variance, where $N_{0}$ is the noise power spectral density (PSD) and W denotes the system bandwidth; * denotes the convolution operation. $h_{VLC}\left(t\right)$ is the optical channel response obtained by the simulation (the nonlinear effect of LED is ignored here).

At the receiver, the captured optical signals are converted to parallel streams and the cyclic prefix is removed. Then, the N points fast Fourier transform (FFT) is applied to the output. The least-square channel-estimated method is applied to estimate the VLC channel state information, and channel equalization is used to equalize the effect of multipath channels. The specific TX and RX modules can be observed in [24,25]. The simulated parameters in this paper are listed in the Table 2.

**Table 2:** The parameters of DCO-OFDM system.

Parameters	Value
QAM modulation order	64
Number of IFFT/FFT ($N_{s}$)	128 [25]
Number of pilots ($N_{p}$)	4 [26]
Information symbols($N_{u}$)	48 [26]
Cyclic prefix of length ($N_{cp}$)	32 [26]

## 3. Analysis of Channel Characteristics

### 3.1. Channel Impulse Responses

The output file, including the information on the received rays (e.g., received power and the path lengths), is generated by the non-sequential ray-tracing tool. By using this information, the channel impulse response which was processed in Matlab${}^{®}$ can be expressed as [27]:

(2) $$h\left(t\right)=\sum_{i=1}^{N}P_{i}\delta \left(t-\tau_{i}\right),$$

where *N* is the number of rays received by PD, $P_{i}$ denotes the received power of the $i^{th}$ ray, δ(t) is the Dirac delta function, and $\tau_{i}$ is the propagation time of the $i^{th}$ ray.

**Figure 2:**
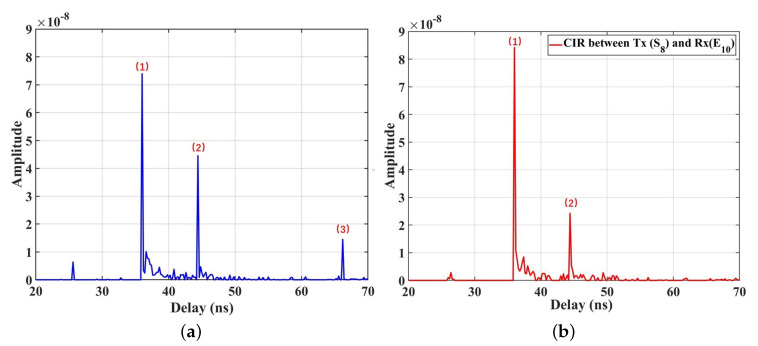
(**a**) NLOS components of CIR for PD $D_{10}$; (**b**) NLOS components of CIR for PD $E_{10}$.

The CIRs under the single TX ($S_{8}$) case are investigated, which PD $D_{10}$ (Road 1) and $E_{10}$ (Road 2) are selected to analyzed. According to Figure 1b and the previous simulation parameters, the LOS distances from these two points to the TX ($S_{8}$) are equal (6.9 m). With which they divide by the speed of light ($3\times 10^{8}$ m/s), the propagation delay of the LOS component is 23 ns. Hence, only the NLOS components of CIRs for the IIoT scenario are drawn in Figure 2, for $D_{10}$ and $E_{10}$, respectively. It is observed that the delays of the first and second peaks (36.0 ns and 44.4 ns) in the Figure 2a are equal to those in the Figure 2b, which are close to the delay of the LOS component. Hence, it can be inferred that those mainly come from the same main reflectors (equipment group B and the floor) shared for both two PDs in Figure 1b. Furthermore, we also find that CIR for $D_{10}$ have more peaks than those for $E_{10}$, which can also be found at other points, i.e., the third peak (66.2 ns) in Figure 2a. This result indicates that the light rays can be received at $D_{10}$ through the reflections of more scatters surrounding Road 1 but only a few reflections by specific scatters can reach the $E_{10}$ in Road 2. It can be explained by the density of surrounding objects. As depicted in Figure 1, the scatter density surrounding $D_{10}$ is larger than that of $E_{10}$, i.e., the more light rays are received. Hence, the third peak in Figure 2a is generated by composing the stronger multipath components (MPCs).

In addition, the VLC channel in the IIoT scenario presents a larger delay fluctuation (70 ns) than that of the VLC channels (20 ns) of the conference room in the [15], which can be explained by the complexity of IIoT scenarios. As discussed in [17], the IIoT scenarios with large physical size metal machine tools make the reflected light rays take longer paths to the RX. Hence, the larger delay of the peak shown in the CIR can be observed.

### 3.2. Optical Path Loss

A VLC-based IIoT channel is characterized by OPL, which can describe the decaying in the received power of the optical signal during its propagation through space.

It can be expressed as:

(3) $$OPL=-10\log_{10}\;(\int_{0}^{\infty }\sum_{i=1}^{N}h_{i}\left(t\right)dt),$$

where the $h_{j}\left(t\right)$ denote the individual optical CIR between the $i^{th}$ LED and the PD, and *N* is the number of rays received by PD. In the realistic environment, the optical path loss is highly related to the environment [6], which requires a model to reflect the actual propagation characteristics. We can model a path loss based on these data samples with the widely used floating-intercept (FI) and close-in (CI) model. The FI and CI model can be expressed as:

(4) $$PL_{FI}=\alpha_{PL}+10\beta_{PL}\log_{10}\left(d\right)+X_{\sigma }^{FI},$$

(5) $$\begin{array}{c} {\mathrm{PL}}^{\mathrm{CI}}\left(f,d\right)=20\log_{10}\left(\frac{4\pi d_{0}f}{c}\right)+10n_{CI}\log_{10}\left(\frac{d}{d_{0}}\right)+X_{\sigma }{}^{\mathrm{CI}}\;\mathrm{for}\;d\geq d_{0},\;d_{0}=1\;m, \end{array}$$

where $\alpha_{PL}$ is the floating intercept, $\beta_{PL}$ and $n_{CI}$ is the path loss coefficient, and $X_{\sigma }^{FI}$ is a zero-mean Gaussian variable with a standard derivation σ representing the shadowing. For VLC channels, the CI model cannot be applied to model the OPL in VLC bands, because this model requires a specific frequency (i.e., f in the CI model), while the LEDs work with mixed wavelengths. Furthermore, concerning the works about the indoor VLC channel modeling [17], the floating-intercept (FI) model shows a good performance to characterize the distance dependence of OPL. Therefore, we choose the FI model to fit the OPL for VLC channels in IIoT scenarios.

**Figure 3:**
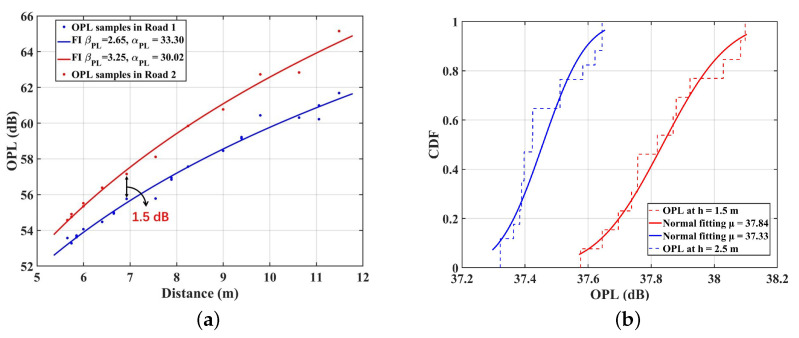
(**a**) The distance dependence of OPL under single TX case; (**b**) the CDF of OPL under the multiple TX case in Road 1.

The OPL under the single TX ($S_{8}$) case is investigated, and Road 1 and Road 2 are selected to analyze it. Figure 3a shows the OPL fitting results with the FI model for VLC channels. The OPL at $D_{10}$ is 1.5 dB smaller than that at $E_{10}$, and we can obtain the same results at other points in this scenario. This is within expectation because of the density of object deployment. As the previous analysis, the objects surrounding Road 1 are denser than that in Road 2, contributing to the stronger specular and diffuse reflection, i.e., more MPCs can be received by the RX. Hence, the total received power is larger and OPL is smaller in $D_{10}$. Furthermore, the PLE of the FI model in Road 2 ($\beta_{PL}$ = 3.25) is larger than that of Road 1 ($\beta_{PL}$ = 2.65). This indicates that the light signals will suffer a faster decay in Road 2 than that in Road 1 with the increasing propagation distance. The differences can also be explained by the environmental configuration around the road. As observed in Figure 1b, in Road 1 with dense machine deployment, the NLOS components are mainly contributed by reflections from the floor and nearby dense machines. However, when machine deployment becomes sparse, the number of reflected rays from nearby devices becomes less, resulting in a weaker received optical power. Hence, the faster decay in the OPL occurs. In addition, compared to the VLC indoor conference model parameter in [15], which is 2.03, the results in this paper have a larger PLE. This can be explained by the unique character of the IIoT scenario. The size of the scenario is larger than that in [15]. The reflected light rays should take longer paths to the RX. Moreover, the light rays split into many random rays and they are reflected in all directions when interacting with the machine with a high specular reflectivity [15]. Finally, only small rays can reach the RX, i.e., a larger PLE can be observed. In addition, the OPL in [28] is also examined by considering LOS and NLOS links. The computed values of the PLE (n) are 1.2 for LOS and 1.4 for the NLOS sub-gallery link, respectively. It can be observed that the PLE in our paper (3.25 and 2.65) is larger than that in [28]. Compared to the physical size of the sub-gallery link (8 × 10 m${}^{2}$), the large physical size is set in the simulation environment (10 × 30 m${}^{2}$). As discussed in [29], the longer link spans means that the light rays take long paths to the RX, i.e., it is a higher OPL that affects the system’s performance. Furthermore, a multitude of reflective surfaces in the IIoT scenario can also lead to fading due to multi-path propagation [30].

Meanwhile, we investigate how the different user heights affect the OPL for VLC channels in IIoT scenarios. The FI model is no longer suitable for fitting the OPL due to the random nature of the light under multiple TX cases. As a result of extensive simulation studies, the normal distribution is used to fit the OPL data [14]. Figure 3b gives the cumulative distribution function (CDF) fitting line of the OPL samples for the VLC channels in Road 1. The mean value of OPL (μ = 37.33) at h = 2.5 m is smaller than that at h = 1.5 m (μ = 37.84). This result indicates that the OPL increases when the user is set to be lower. The difference is due to the occlusion from surrounding objects, whose average heights are set to 2 m. At h = 1.5 m, most of the objects are higher than the users. The reflective multipath is blocked by the objects, resulting in heavy shadow fading [31]. Hence, the received optical power is weakened. Meanwhile, at h = 2.5 m, the surrounding objects are generally lower than the users. There is less occlusion to the light rays, and more multipaths can be received by the RX, i.e., smaller OPL at h = 2.5 m. Furthermore, the mean values of the OPL fitting results for VLC channels in the IIOT scenario are larger than that for the indoor hospital (60–70 dB) in the [14]. This can be explained by the number of LEDs deployed on the ceiling. Compared with the number of LEDs (15) setting in the [14], the IIoT environment in this paper is deployed with 21 LEDs, i.e., contributing much more MPCs received by the RX. Hence, the OPL is smaller in the IIoT scenario.

### 3.3. RMS DS

Another essential channel characteristic is the RMS DS, which is a metric presenting the temporal dispersion of the multipath transmission. Furthermore, the inter-symbol interference may be caused by large DS. It is defined as:

(6) $$\begin{array}{c} \tau_{RMS}=\sqrt{\frac{\int_{0}^{\infty }{\left(t-\tau_{0}\right)}^{2}h\left(t\right)dt}{\int_{0}^{\infty }h\left(t\right)dt}}, \end{array}$$

where $\tau_{0}$ denoted the mean delay:

(7) $$\begin{array}{c} \tau_{0}=\frac{\int_{0}^{\infty }th\left(t\right)d\left(t\right)}{\int_{0}^{\infty }h\left(t\right)d\left(t\right)} \end{array}$$

**Figure 4:**
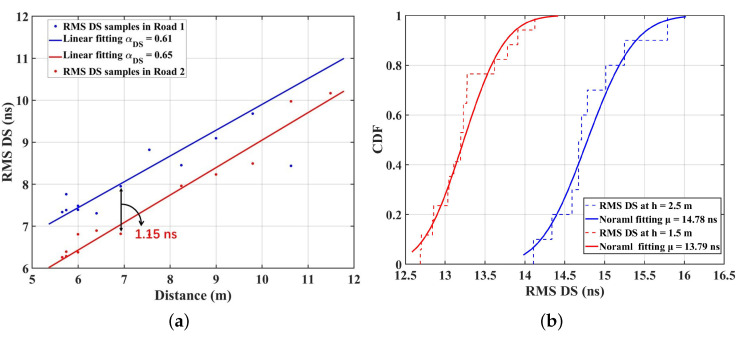
(**a**) The distance dependence of RMS DS samples under single TX case; (**b**) the CDF of RMS DS under the multiple TXs’ case in Road 1.

The RMS DS has often been reported to increase with distance and to model the RMS DS; the distance-dependent model is selected as one of the candidate models in 3GPP [7]. The linear model is often used to fit the RMS DS in existing research [31,32]. Therefore, to model the RMS DS samples when the single LED ($S_{8}$) is only considered as the TX, we can model the RMS DS with the linear model to describe the distance–dependence, as shown in [33], which can be expressed as:

(8) $$\begin{array}{c} \tau =\alpha_{DS}\cdot d+\beta_{DS}+X_{\sigma }^{DS}, \end{array}$$

where $\alpha_{DS}$ and $\beta_{DS}$ are the slope and intercept of fitting curves, and $X_{\sigma }^{DS}$ is the standard deviation between the RMS DS and fitting curve. Figure 4a presents the RMS DS for VLC channels calculated by (6), and the fitting results with the linear model in Road 1 and Road 2. The slopes of the fitting models are 0.61 and 0.65, respectively, for Road 1 and Road 2. Obviously, under the single TX case, the positive slope indicates that the RMS DS increases with the propagation distance. As the RX is farther from the TX, the surrounding reflection environment becomes more complicated, resulting in more MPCs received and greater RMS DS. Furthermore, the RMS DS at $D_{10}$ is 7.95 ns, which is slightly larger than that at $E_{10}$ (6.80 ns). As in the previous analysis, the more dense surrounding objects contribute much more MPCs. This explanation can also be demonstrated by the NLOS components of CIRs in Figure 2. CIRs for $D_{10}$ have more peaks with larger delay (70 ns) than those for $E_{10}$ (50 ns), which can also be observed at other points in the two roads. Hence, the RMS DS is larger. Moreover, the RMS DS range for VLC channels is larger (6–11 ns) compared to that observed in the underground mining channel (2–8 ns). This can be attributed to the unique characteristics of the IIoT scenario examined in this study, which has a larger scenario size than [34]. Additionally, industrial environments usually have highly reflective surfaces such as metal fixtures and equipment [29]. The simulated environment in this paper with objects deployed is more complex compared to the work areas in [34]. As a result, a larger number of MPCs are generated during the propagation from the TX to the RX, leading to a larger RMS DS in VLC channels. Furthermore, the VLC channel in the paper presents a larger delay fluctuation (70 ns) than that of the VLC channels (30 ns) of the underground mines in the [34], which can also be explained by the complexity of IIoT scenarios.

Moreover, the RMS DS can be fitted well by a normal distribution. Under the multiple TXs case, Figure 4b gives the CDF fitting line of the RMS DS samples with different heights’ RX in Road 1. The mean value of RMS DS (μ) at h = 1.5 m is 13.79 ns, which is smaller than that of 14.78 ns at h = 2.5 m. This can be explained by the blocking effect of objects. As the height of the user is lower than the average height of objects, the light signals are more likely to be obstructed by objects, e.g., manufacturing equipment, assembly lines, and forklifts. The blocking will lead to fewer MPCs for users at h = 1.5 m than 2.5 m, i.e., the RMS DS is reduced. In addition, this paper shows larger RMS DS than that in [13] (6–11 ns) under multiple TXs. This is mainly because the IIoT scenario has a larger physical size and much more complicated equipment, while the living room in [13] is small and empty. The light rays generated by the multiple LEDs will experience complicated reflections and be reflected in all directions, so that more MPCs reach the RX, resulting in the larger RMS DS for VLC channels in this paper.

### 3.4. Angular Spread

**Figure 5:**
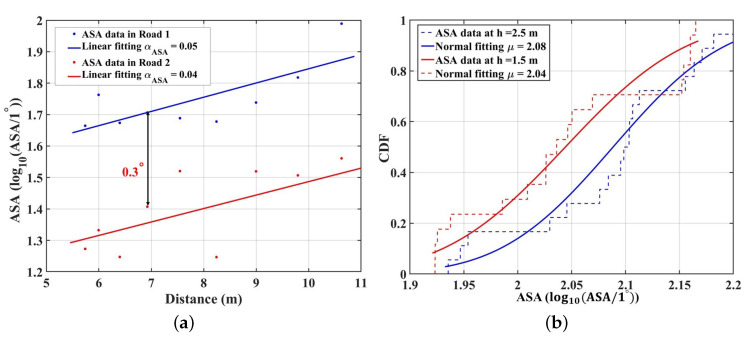
(**a**) The distance dependence of ASA samples under single TX case; (**b**) the CDF of ASA under the multiple TXs’ case in Road 1.

Studying the spatial angular properties of the channel helps to understand the distribution of objects in the scenarios’ propagation mechanism of the signal. The angular spread is a key parameter to describe the directions of MPCs [35]. The AS of arrival (ASA) can be calculated by [36]:

(9) $$\sigma_{ASA}=\sqrt{-2(\left|\frac{\sum_{r=1}^{R}exp\left(j\phi_{r}P_{r}\right)}{\sum_{r=1}^{R}P_{r}}\right|)},$$

where $\phi_{r}$ and $P_{r}$ are the angle in radians and power of the *r*-the path. The distance dependence of ASA in the office and corridor is fitted by the linear model [31]:

(10) $$ASA\left(\log_{10}\left(ASA/1^{\circ }\right)\right)=\alpha_{ASA}\cdot d+\beta_{ASA}+X_{\sigma }^{ASA},$$

where $\alpha_{ASA}$ and $\beta_{ASA}$ are the slope and intercept of fitting curves, and $X_{\sigma }^{ASA}$ is the standard deviation between the AS and fitting curve. Figure 4a presents the real number results for ASA data calculated by (9) and (10), and the fitting results with the linear model in Road 1 and Road 2. The positive slopes of the fitting models (0.04 and 0.05) indicate that the ASA is proportional to the propagation distance under the single TX case. At short propagation distances, the LOS component occupies an absolute power ratio, and the proportion of NLOS components is small. Hence, the received components mainly come from the LED source. However, the LOS component occupies an absolute power ratio, and the proportion of NLOS components is pretty small. However, as the propagation distance increases, the proportion of NLOS components also increases and cannot be overlooked. The distributed signal with power amplitude fluctuations from all directions leads to a larger ASA. Furthermore, the ASA at $D_{10}$ is $0.3^{\circ }$ larger than that at $E_{10}$. The RX surrounded by the denser complicated objects will receive more MPCs, leading to more NLOS components. Hence, the ASA is larger. Furthermore, the ASA can be fitted well by a normal distribution. Under the multiple TXs’ case, Figure 5b gives the CDF fitting line of the ASA samples with different heights’ RX in Road 1. To confirm our results for the fitting of actual and normal graphs, the statistical test function in the Matlab, i.e., the Kolmogorov–Smirnov test (KS-test), is used to decide whether the ASA data follow the Normal distribution. The result obtained is zero, indicating that VLC cluster numbers follow the Normal distribution. The mean value of ASA (μ) at h = 1.5 m is 2.04, which is smaller than that of 2.08 ns at h = 2.5 m. This can also be explained by the blocking effect of objects. Some MPCs received at the RX with a 1.5 m height will be blocked by the surrounding objects, which are set to be higher than 1.5 m, i.e., the NLOS components are reduced. Hence, the mean value of the ASA in the 2.5 m is larger. Furthermore, the fitted mean value of the ASA at 1.5 m is 2.08, which shows a larger value compared to the measured ASA in [37] (1.46). It is mainly because the IIoT scenario is larger and more complicated than the conference room in [37]. The reflecting and scattering multipath can be received in this paper, which makes the ASA larger.

According to the above analysis, it can be found that the degree of the time domain and spatial domain dispersion (RMS DS and ASA) for VLC channels in this paper is higher than that in the typical rooms of the related works. These differences can be explained by the specificity of the IIoT scenarios, including the large physical size and the abundance of metal scatterers deployed. The complex VLC channel characteristics caused by the features of IIoT scenarios make the VLC system require a good robustness with optimization algorithms for overcoming inter-code interference.

## 4. Link Adaption Method to Optimize the Multipath Interference

As observed from Figure 3, Figure 4 and Figure 5, the channel characteristics vary significantly based on the user location. Furthermore, the large time-domain dispersion (RMS DS) in the IIoT scenario will lead to severe ICI. To solve this problem, a two-step approach to optimizing the multipath interference with distance variation is further proposed. The algorithm details are shown in the Algorithm 1. Firstly, the greedy algorithm is used to select the suitable LED index to maximize the SNR. The received power from each TX to the RX is first calculated. Then, the received power matrix is sorted in descending order ($P_{sort}$). Furthermore, the sequence is accumulated ($P_{cum}$). Then, filter out the LED index that contributes 95 percent of the total received power ($P_{LED}$). The optimal index and the maximum received power ($P_{r}$) are further selected to meet the maximum SNR. The maximum SNR can be calculated by Equation (11) according to the noise power and index of active LEDs. Moreover, compared with the brute-force search algorithm (O(N!)) in the [13], the greedy algorithm in this paper has a lower algorithmic complexity (O(NlogN)).

(11) $$SNR=\frac{P}{\left|\phi \right|\sigma_{N}^{2}}{\left|\sum_{i\in \phi }H_{i}\right|}^{2},$$

where P is the total information power, $H_{i}$=$\int_{0}^{\infty }h\left(t\right)d\left(t\right)$, ϕ is a vector including the index of active LEDs, |ϕ| denotes the number of active LEDs, and $\sigma_{N}^{2}$ is the noise power. Although the suitable LED index is selected to maximize the SNR, the number of LEDs has not been significantly reduced, i.e., CCI problems should be solved. In this paper, the delay adaptation method in the [19] is used. This method can control the time of transmitting the effective signal so that the LOS components from variable transmitters arrive at the receiver side at the same time. The specific method is listed in the Algorithm 1 Setp 2.

**Figure 6:**
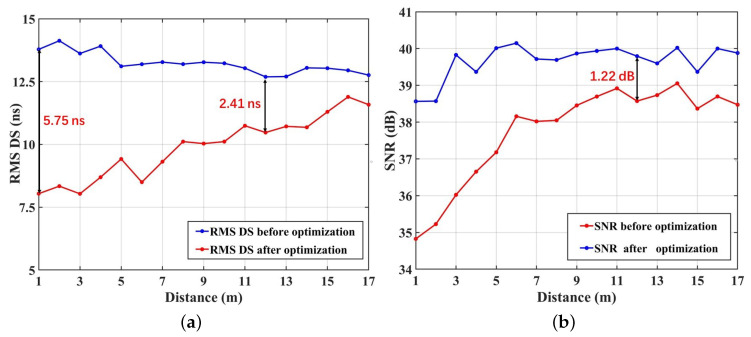
(**a**) Comparison of RMS DS before and after optimization; (**b**) comparison of SNR before and after optimization.

**Figure 7:**
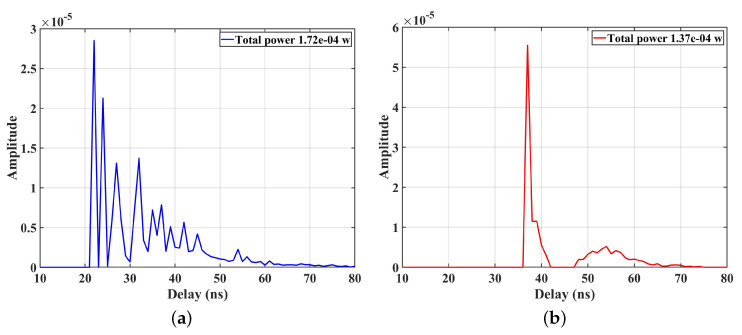
(**a**) The CIR before optimization; (**b**) the CIR after optimization.

**Figure 8:**
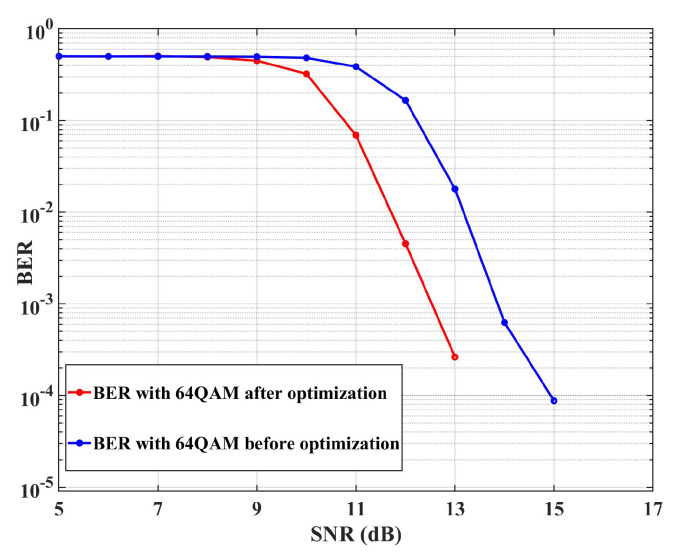
The BER performance of the DCO-OFDM system in the IIoT channel.

After the data processor based on the two-step approach to optimizing the multipath interference, four parameters, including the SNR, the RMS DS, the CIRs, and the BER of the DCO-OFDM system, are simulated to verify the performance of the link adaption method. The results are drawn in Figure 6, Figure 7 and Figure 8. Figure 6a shows the RMS DS of the user walking from the wall side to the center in Road 1, and the Figure 6b gives the SNR before and after optimization. The difference for RMS DS before and after optimization is 2.41 ns in the $D_{10}$, and the difference for SNR is 1.22 dB. Furthermore, the closer the user is to the center of the room, the smaller range can be optimized. This is mainly because as the distance increases, the interval of LEDs that can be selected for optimization increases, i.e., the number of optimized LEDs decreases, resulting in a larger RMS DS. Figure 7 gives the CIR superimposed by the LOS and NLOS components before and after optimization in Road 1 under multiple TXs’ cases. It can be found that the received power at $D_{10}$ decreases from $1.72\times 10^{-4}$ before optimization to $1.37\times 10^{-4}$ after optimization. Furthermore, after optimization, the number of the main multipath in the CIR decreased from 9 to only 1. This indicates that the system can significantly reduce the impact of multipath fading based on the reduced receiving power by using the multipath optimization method in the paper.
**Algorithm 1:** A two-step approach to optimizing multipath interference**Step** **1:**Adaptive LED selection method
Descending order of power $\left[P_{sort}\right]\leftarrow H_{1\times N}$Cumulative sum power $P_{cum}\leftarrow cumsum\left(P_{sort}\right)$Pick the index that meets power requirements$P_{LED}=P_{cum}\left(0.95\times \sum_{r}P_{cum}\right)$Select the optimal index that meets the maximum SNR$P_{cumopt}\leftarrow cumsum\left(P_{LED}\right)$$P_{r}^{2}=max\left(\frac{P_{LED}^{2}}{1:len(P_{cumopt})}\right)$
**Step** **2:**Optimization for co-channel interference
Calculate the Relative delay of LOS components:$Delay_{rev}$=$max\left(\tau_{LOS}\right)-\tau_{LOS}$;Add the Relative delay for CIRs from LEDs:$Delay_{1\times N}^{new}$=$Delay_{1\times N}^{origin}$+$Delay_{rev}$;Output SNR based on Equation (11) and CIR


Furthermore, to evaluate the system performance of DCO-OFDM in the IIOT multipath channel before and after the optimization, the simulation platform is built in MATLAB based on the previous parameters. Figure 8 gives the BER results with 64 QAM orders in the cases of the CIRs in Figure 7. It is found that the larger the SNR for 64QAM in the DCO-OFDM system, the DCO-OFDM system working in Figure 7b shows a better performance than that in Figure 7a. This indicates that the system performance of DCO-OFDM is significantly improved after the multipath interference optimization. The finding can also be found in the [25]. This is because the system performance is dominated by the frequency-selective nature of the channel (BER due to the channel fades). As the CIR demonstrated in Figure 7, the multipath before the optimization is more than those after optimization. When the transmitted data are convolved with the CIR ($h_{VLC}\left(t\right)$) by using the (1), they are changed by the multipath components with the higher power. The correlation of the training sequence as a time synchronization will be destroyed, resulting in the inability to execute accurate coarse and fine synchronization. Therefore, the multipath components will interfere with the demodulation constellation diagram, resulting in the deterioration of BER. Furthermore, the minimum value of the BER is approximately equal to zero at the SNR of 17 dB, which is sufficient for reliable communication.

## 5. Conclusions

In this paper, the VLC channel in the IIoT scenario is investigated based on the ray-tracing simulation. The large-scale fading and multipath-related characteristics are analyzed and modeled. The modeling results indicate that the OPL, the RMS DS, and AS are proportional to the propagation distance under the single TX. Moreover, due to the complexity of the scenario, it can also be found that the degree of the time domain and spatial domain dispersion in the IIoT scenario is higher than that in the indoor scenarios (conference room and office). Furthermore, the VLC channel characteristics are significantly affected by the density of objects and the height of the user. The differences for the OPL and RMS DS are, respectively, 1.5 dB and 1.15 ns at the RX with the various objects’ densities surrounding it. Moreover, the occlusion of the surrounding objects makes OPL larger and RMS DS smaller when the height of users is lower than surrounding objects. In addition, motivated by the fact that channel characteristics vary significantly based on the user location and multipath interference to be solved, an adaptive LED selection combined with the delay adaptation technique is further proposed. Then, to verify the performance of the proposed method, four parameters are simulated, including the SNR, RMS DS, CIRs, and the BER performance of the DCO-OFDM system. It can be found that there is a significant decrease in the RMS DS and the BER of the DCO-OFDM system after the optimization. Thus, these results indicate that our proposed link adaption method can be effective for multipath interference optimization. Furthermore, the minimum value of the BER is zero at the SNR of 17 dB, which is sufficient for reliable communication.

## Figures and Tables

**Table 1:** Parameters of simulation setup.

Item	Parameter	Value
Specifications of scenario	Dimensions	30×10×8 m${}^{3}$
	Number of sources	21
	Average height of machine	2 m
Coating materials of objects	Walls, ceiling, and floor	Plaster [18]
	Manufacturing equipment	Cu
	Assembly lines, forklifts	Al, Fe
Scatter fraction	Walls, Ceiling, Floor	0.8
	Manufacturing equipment	0.2 [11]
	Other objects	0.5 [11]
TX	Model of LED	Lambert
	Optical power of each LED	2 W
	Relative minimum Intensity	10${}^{-3}$ [18]
Channel	Delay resolution	0.2 ns [15]
RX	Type of receiver	Detector Polar
	Radial size	10 mm

## Data Availability

Not applicable.
